# Groundwater nitrate pollution and climate change: learnings from a water balance-based analysis of several aquifers in a western Mediterranean region (Catalonia)

**DOI:** 10.1007/s11356-018-1859-8

**Published:** 2018-04-11

**Authors:** Josep Mas-Pla, Anna Menció

**Affiliations:** 1 Institut Català de Recerca de l’Aigua (ICRA), Girona, Spain; 2 Grup de Geologia Aplicada i Ambiental (GAiA), Centre de Recerca en Geologia i Cartografia Ambiental (Geocamb), Dept. de Ciències Ambientals, Universitat de Girona, Girona, Spain

**Keywords:** Groundwater, Nitrate, Climate change, Scarcity, Sustainability

## Abstract

Climate change will affect the dynamics of the hydrogeological systems and their water resources quality; in particular nitrate, which is herein taken as a paradigmatic pollutant to illustrate the effects of climate change on groundwater quality. Based on climatic predictions of temperature and precipitation for the horizon of 2021 and 2050, as well as on land use distribution, water balances are recalculated for the hydrological basins of distinct aquifer systems in a western Mediterranean region as Catalonia (NE Spain) in order to determine the reduction of available water resources. Besides the fact that climate change will represent a decrease of water availability, we qualitatively discuss the modifications that will result from the future climatic scenarios and their impact on nitrate pollution according to the geological setting of the selected aquifers. Climate effects in groundwater quality are described according to hydrological, environmental, socio-economic, and political concerns. Water reduction stands as a major issue that will control stream-aquifer interactions and subsurface recharge, leading to a general modification of nitrate in groundwater as dilution varies. A nitrate mass balance model provides a gross estimation of potential nitrate evolution in these aquifers, and it points out that the control of the fertilizer load will be crucial to achieve adequate nitrate content in groundwater. Reclaimed wastewater stands as local reliable resource, yet its amount will only satisfy a fraction of the loss of available resources due to climate change. Finally, an integrated management perspective is necessary to avoid unplanned actions from private initiatives that will jeopardize the achievement of sustainable water resources exploitation under distinct hydrological scenarios.

## Introduction

Groundwater nitrate pollution is a ubiquitous worldwide problem that has been in the managers’ as well in the researchers’ agenda for decades (e.g., Galloway et al. 2008; Sutton et al. 2011; Rosenstock et al. 2014). Multiple efforts have been devoted to improve agricultural practices so nitrogen leaching due to fertilization is minimized (Cameron et al. 2013; Beaudoin et al. 2005), to track its movement through the unsaturated zone and its migration beneath the water table (Baram et al. 2017; Dimitriou and Moussoulis 2010; Molénat and Gascuel-Odoux 2002), to analyze biogeochemical processes so denitrification rates can be identified and induced aquifer clean-up be subsequently applied to remove nitrate from groundwater (Böhlke 2002; Rivett et al. 2008), and, finally, to treat polluted groundwater once withdrawn from the aquifer so its nitrate content is reduced before entering the water supply systems (Seidel et al. 2011). Moreover, legislation has been issued to cope with these problems and oblige those who are responsible to reduce such impact and/or face legal prosecution (i.e., the Nitrates Directive, 1991/676/EEC; and the Water Framework Directive, 2000/60/EEC).

Despite all these efforts, present nitrate concentrations in groundwater reflect the impact of decades of nitrogen inputs and its persistence in the subsurface (e.g., Paradis et al. 2017). In many aquifers, dilution has been one of the major processes that have diminished nitrate concentration (Altman and Parizek 1995; Hoffman and Canace 2004). Mixing between polluted water resources and less-polluted groundwater effectively reduces this environmental pressure. In the subsurface, natural and pumping induced mixing from different aquifer levels also has a dilution effect. In particular, mixing in boreholes due to the exploitation of several aquifer levels produces dilution by averaging nitrate mass fluxes, which in many cases is not enough to reduce nitrate concentration below the drinking standard values. However, mixing cannot be understood as a complete positive effect, as non-polluted groundwater resources are also at risk to reduce their quality. In intensively exploited aquifers, well fields induce an overall mixing process that affects the general quality of the regional subsurface resources.

Dilution, at the end, relies on the input of non-polluted water fluxes. Assuming that the aquifer has such inputs (i.e., through rainfall, stream and/or mountain front recharges), a decrease on the amount of “clean” fluxes definitely reduces the mixing capacity and, therefore, the dilution effect. In many regions on Earth, and in particular in western Mediterranean areas, climate change will represent a shift towards drier conditions (e.g., IPCC 2014), and consequently a diminution of the aquifer dilution capacity in relation to its aquifer turnover or mean residence time (Kazemi et al. 2006). Since hydrogeological resources are highly influenced by human pressures, the effect of climate change upon groundwater bodies must be considered from distinct perspectives. Both climate and human pressures define global change aspects. Such approach can be focused to investigate the persistence of nitrate in groundwater by considering all the processes that, once affected by global change, will modify the tendency of nitrate concentrations and that of groundwater quality.

The goal of this contribution is to explore the effects of global change—that is, those derived from climate variations and human pressures—upon the fate and persistence of nitrate in groundwater at a regional scale and in the long run. It is our belief that such a comprehensive analysis supports the worth of future actions on nitrate management to ensure the sustainability of water resources. To conduct such objective, a water balance approach, which conceptually analyzes the variation that each component of the hydrological cycle will experience under new climatic scenarios and similar water uses, is used to address the effect of climate change on the groundwater nitrate content. Hydrological, environmental, and socio-political concerns are discussed so distinct perspectives are considered in the evaluation of such interdisciplinary issue (Holman 2006).

During the last decade, several studies have characterized the extent of nitrate pollution and the occurrence of denitrification processes from a hydrogeological and hydrogeochemical perspectives in distinct aquifers of Catalonia (NE Spain). Five out of the ten aquifers declared vulnerable to nitrate pollution in these region, according to EU directives, have been investigated, and the results published in distinct scientific journals, PhD dissertations or technical reports (references detailed in the “Methodology” section).

**Fig. 1:**
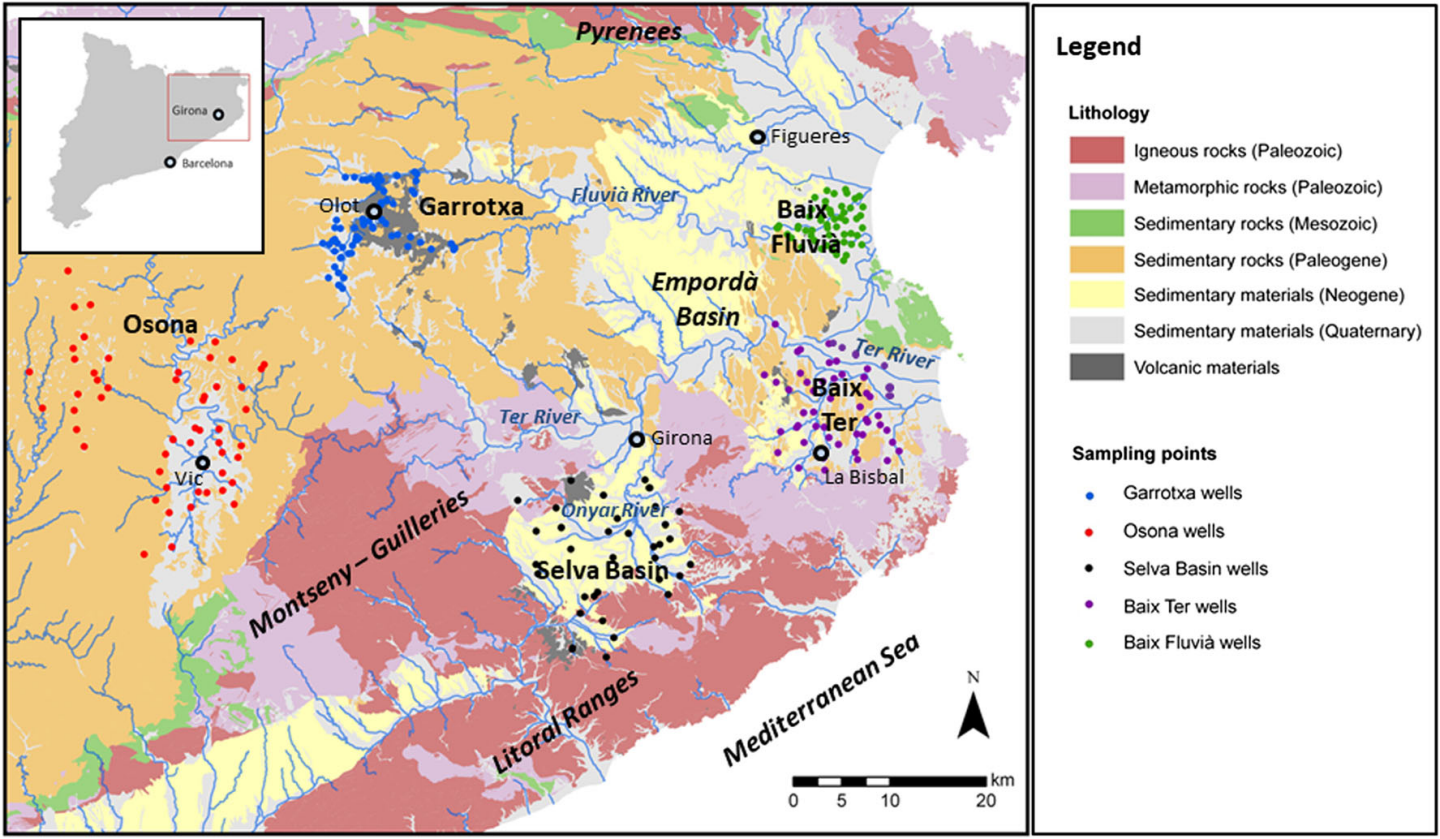
Geographical locations and geological setting of the studied areas (Catalonia, NE Spain). Geological background form ICGC (2017)

In this contribution, we use these selected aquifers in Catalonia as paradigmatic cases to analyze the effect of climate change on nitrate concentration for a western Mediterranean climatic setting (Fig. 1). For each aquifer, their hydrogeological characteristics, the extent of the pollution and denitrification processes, the rate of groundwater exploitation and its final use, as well as the occurrence of alternative water resources for human uses. Lastly, the pressures upon groundwater quality (e.g., fertilization, wastewater infiltration) are described as a basis to illustrate potential hydrologic behaviors under future hydrologic scenarios (Mas-Pla 2018). This analysis provides thus a qualitative evaluation for the future availability of suitable groundwater resources in these aquifers on the light of the expected water reduction related to global change, as defined by the climatic scenarios for the western Mediterranean area and land use changes.

A paradigmatic study on the impact of climate change on future nitrate concentrations in groundwater of the UK by Stuart et al. (2011) summarizes potential changes on the hydrological processes that will affect nitrate concentration using a source–pathway–receptor framework. These authors concentrate on three main questions to understand the nature of climate change impact on groundwater nitrate concentrations:

i. What are the likely changes to agricultural practices and how may these affect nitrate leaching from the soil zone?
ii. What are the likely changes to groundwater recharge mechanisms and groundwater levels?
iii. What are the likely changes to nitrate concentrations in groundwater and the consequent impact on groundwater receptors?

Assuming that few changes will occur on agricultural activities in the next decades to 2050 (as discussed hereafter), questions ii and iii are those of most interest, and they will be addressed in this study using a water balance approach. We assume that the principal nitrogen input will continue to derive from fertilization, whether using organic manure or slurry, chemical fertilizers, and/or sewage slurries, and this should remain alike for the next decades. In many regions, such as Catalonia, the use of livestock manure as fertilizer will stay as the main nitrogen source used in crops as a means to reuse this by-product. At a regional level, best management practices are one of the driving factors to reduce nitrate from agricultural origin in groundwater. Point sources inputs of domestic and industrial origin are also relevant (Ducharne et al. 2007), yet these are not relevant as poles of groundwater nitrate pollution in the studied aquifers.

## Methodology

This study deals with the effect of future climatic scenarios (i.e., horizons 2021 and 2050) on groundwater quality based on already existing hydrogeological knowledge of several nitrate polluted aquifers. Data from each aquifer have been reported by the following references: Osona region (Vitòria et al. 2008; Otero et al. 2009; Menció et al. 2011b; Boy-Roura et al. 2013a); the Selva Basin (Folch et al. 2011; Menció and Mas-Pla 2010; Menció et al. 2012; Puig et al. 2013), the Garrotxa area (Bach 2015, personal communication), the Baix Ter aquifer (Mas-Pla et al. 1998; Puig et al., 2017), and the Baix Fluvià aquifer (Montaner et al. 1998; Boy-Roura et al. 2018). Pollution levels in most of these aquifers were summarized and discussed by Menció et al. (2016).

Climate predictions allow estimating the subsequent modifications of the water budget for each hydrogeological system that will follow. So, climate data for the above-mentioned horizons were calculated after a downscaling process from global circulation models by Calbó et al. (2016). This reference provides the averaged temperature increment and rainfall variations for different geographical/climatic areas in Catalonia. Water budgets in the hydrographic basins of Catalonia, with special interest of those corresponding to the studied aquifers, were estimated by Mas-Pla et al. (2016) using the temperature and precipitation projections by Calbó et al. (2016) and the percentage of land use to compute actual evapotranspiration as determined by Zhang et al. (2001). To differentiate between runoff and infiltration terms, we use a map of average recharge based on the chloride mass balance by Alcalá and Custodio (2014).

To quantify the effect of climate change on groundwater nitrate content, an original mass balance approach, based on a lumped-parameter model, provides an evaluation of the impact of changing hydrological conditions on the selected aquifers. The approach presented by Thomann and Mueller (1987; Appendix 1) considers an aquifer with a constant water storage (*dV*/*dt* = 0), under a constant flow, *Q*, and a variable nitrate mass input, *W*(*t*). The change of the solute concentration over time, *c*(*t*), in the aquifer during a given time period is given by

$$ V\frac{dc}{dt}=W(t)- Qc- KVc $$

This model assumes that diffuse nitrogen inputs, *W*(*t*), on the aquifer surface are completely mixed horizontally and vertically. This assumption seems reasonable based on the large extent of the agricultural land use compared to the aquifer thickness, especially on thin alluvial systems. Degradation, as due to denitrification processes, is represented by a first-order decay factor embedded as a reactive term, *K*. Such a gross approximation provides a quick and useful portrait of the system behavior avoiding the elaboration of complex numerical flow and transports models. To solve the former equation, aquifer dimensions were estimated and porosity taken accordingly to the dominant lithology (Appendix 2). Natural outflow, *Q*, equal to the corresponding inflow to insure no changes in aquifer storage, were estimated in three different ways: (1) using the recharge rate estimated for the present day scenario (2005–2015) in uniform conditions for a period of 50 years; (2) using the recharge rate estimated for the hydrological conditions forecasted for 2050, also considered uniform during the simulation; and (3) assuming that recharge rate changes linearly at annual time steps from present hydrological conditions to those predicted by 2050. This third option implies that the initial concentration in the reservoir will also change annually as the inflow/outflow rate varies. Local water balance data for present and future climatic scenarios are derived from Mas-Pla et al. (2016) estimations, and they are summarized in the next sections.

The total mass of nitrate entering the aquifer considers a generalized application of 170 kg N/ha/year from livestock manure, as permitted by the EU Nitrates Directive. No other nitrogen inputs are considered. It is indeed difficult to assign future fertilization rates as input loads in the mass balance model. Even though future agricultural management will eventually reduce rates, it looks sensible to think that farmers will take the chance to apply as much fertilizer as allowed by law. Nitrogen uptake in each aquifer was calculated as the product of the nitrogen assimilation by each type of crop times the reported local production in 2016 and the total crop area in the considered aquifer. Details are given in Appendix 3. A first-order decay term for nitrate in aquifers due to natural denitrification processes is initially taken as 0.04 year^−1^, which is a lower, cautious value (Carroll et al. 2009).

We herein profit climatic results and field data from existing information, and the outcomes of the nitrate mass balance model to discuss the impact of climate change on nitrate pollution for distinct hydrogeological settings using a water balance approach (e.g., Menció et al. 2010; Fitts 2013, pp. 13–16). In other words, the effects of climate change on the components of the hydrologic cycle and the consequences on groundwater nitrate content are evaluated at the level of hydrological process and under distinct hydrogeological frameworks given by the geological idiosyncrasy of the selected aquifers.

## Summary of hydrogeological, climate, and water balance data

### Hydrogeology and water quality in the considered nitrate vulnerable aquifers

This study considers data from five aquifers in Catalonia, all of them defined as vulnerable zones to nitrate pollution under the present legislation and the water agency management plans (Fig. 1). These aquifers represent distinct hydrogeological environments, so the climate change impact on the water budget can be discussed for different geological circumstances; for instance, it can be expected that stream-recharged unconfined aquifers will respond differently than confined aquifer systems under rainfall recharge variations. In general, nitrate origin is mostly related to fertilization practices using cattle rising manure.

Data from the studied regions are summarized for each aquifer (Table 1); in particular: (1) aquifer type (water table, leaky, or confined); (2) origin of recharge: direct rainfall (representing soil infiltration and, therefore, leaching of sol nutrients and applied fertilizers), interaction with streams (considering discharge regime), and occurrence of recharge from mountain fronts or the basement according to the geological setting; and (3) mean and median nitrate content, percentage of samples showing denitrification, and (4) percentage of denitrification based on the multi-isotopic approach. In all these areas, land use is mainly occupied by agricultural and forested areas, and irrigation relies on groundwater; except in the Baix Ter system where surface water diversion from the Ter River is also used for irrigation.

**Table 1:** Summary of the main hydrogeological and chemical status of the distinct aquifers considered in this study. Data after references cited in the text

	Osona	La Selva	Onyar	Garrotxa	Baix Ter	Baix Fluvià
Aquifer lithology: bedrock, sedimentary infilling, alluvial	B - A	S - A	A	B - A	S - A	A
Recharge origin: local, regional	L - R	L - R	L	L	L - R	L
Aquifer type: unconfined/multilayered/leaky	M	U - M	U	U - M	U - M	U
[NO_3_] (mg/L): mean/median/% > 50 mg/L	165/92/72%	65/59/51%	61/47/45%	35/29/22%	78/67/57%	43/19/22%
δ^15^N_NO3_ (‰): mean/median/% > 15‰^a^	14.0/13.4/28%	10.8/10.4/7%	[No data]	9.9/9.4/6%	12.9/12.2/22%	9.2/9.1/0%
Land use (%): agricultural/irrigated crops/others	A (21%)	A (28%)	A (64%)	A (16%)—mostly forested	A (37%)	A (54%)
Groundwater exploitation: seasonal, non-seasonal	NS (livestock)—S (agriculture)	NS (livestock)—S (agriculture)	NS (livestock)—S (agriculture)	S (agriculture)	NS (urban)—S (agriculture)	NS (livestock)—S (agriculture)
Alternative resources	Ter River (mid-basin)—water reclamation	Water reclamation	Water reclamation	Fluvià River	Ter River (lower-basin)—water reclamation	Fluvià River
Other pressures, hazards	(None)	Local As, F high levels	Over-exploitation	(None)	Seawater intrusion—over-exploitation	(None)

^a^Values of δ^15^N_NO3_ > 15‰ are assumed to be indicative of denitrification processes

**Fig. 2:**
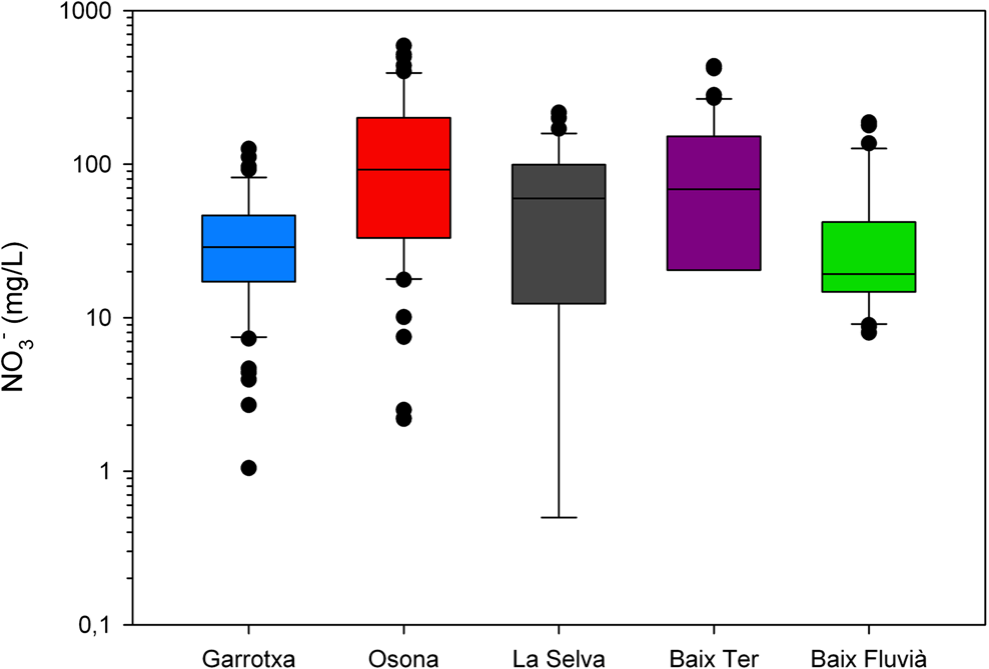
Box plot of the nitrate concentration distribution in each of the studied aquifer systems. Data from the diverse references cited within the text

A brief description of the studied aquifers, based in the above-mentioned references, is provided next (refer to Fig. 1 for geographical and geological setting and to Fig. 2 and Table 1 for their nitrate concentration distributions):

#### Osona aquifer

The Osona hydrogeological system develops on the Tertiary sedimentary rocks in central Catalonia, characterized by a suite of sandstone and marl layers. The fracture network in these consolidated sedimentary rocks controls groundwater flow and infiltration. Surface deposits (regoliths, colluviums) that sustain agricultural activity retain fertilizer inputs that lately infiltrate to the underlying sedimentary layers. Nitrate mean value is 165 ± 18 mg/L, which indicates the occurrence of very high concentrations. The influence of groundwater exploitation forces nitrate polluted water downward through the fractured marls that act as aquitards. After decades of intense (and initially unregulated) fertilization practices, high nitrate concentrations are usual in this area. Denitrification due to sulfate reduction is found in the northwestern and north central areas where pyrite occurs within the marly sediments.

#### La Selva aquifer

This aquifer system consists of the sedimentary infilling of a tectonic depression originated during the Neogene. Sediments originated from the weathering and erosion of the surrounding ranges with igneous and metamorphic rocks and were deposited as alluvial fan systems. Therefore, it is formed by a multilayer aquifer with gravel, sand, and clay layers with a maximum thickness of near 200 m in its westernmost area. Small alluvial aquifers develop along the main streams, yet the maximum groundwater withdrawal originates from the Neogene unit. Fault zones located in its boundaries, as well as in the basement, allow the recharge of the sedimentary aquifer by upward vertical regional flows. These contribute to the potentiometric head recovery of the leaky aquifer layers in winter and spring. Nitrate content is high (mean value 65 ± 10 mg/L), with half of the sampling points above the threshold limit for water consumption. Denitrification is not intense and it has been only recorded in a few samples. Within the Selva aquifer, the case of the Onyar River aquifer has been considered as an example of a thin alluvial, with an intense agricultural activity and high nitrate content (mean concentration 61 ± 7 mg/L).

#### The Garrotxa aquifer

Sampled wells in this aquifer are located in the alluvial-lacustrine sediments that fill the valleys in the volcanic area of La Garrotxa. Volcanic activity, especially, lava flows favourished the sedimentation of alluvial-lacustrine deposits which infilling correspond to the erosion of the sedimentary rocks from the surrounding ranges and to the lapilli and lava fragment eroded from the volcanic materials. The balance between nitrogen inputs and groundwater recharge results in low average nitrate content (mean concentration 35 ± 4 mg/L). Denitrification processes have not been identified in this aquifer.

#### The Baix Ter aquifer

This hydrogeological system includes a Ter River fluvio-deltaic system and the surrounding sedimentary rocks of its southern margin. Stream recharge is relevant in the western zone, while the Ter River acts as a gaining stream in its central and lower reaches. Most of the nitrate concentration is found in the upper unconfined layer of the fluvial system, although also in the wells located in the sedimentary rock formations underlying agricultural areas. High nitrate content is usual, but nitrate occurrence is variably distributed; for instance, concentrations ranged from 6 to 480 mg/L in shallow aquifer, while they seldom achieve the 50 mg/L in the deeper layers. The overall mean concentration value in the shallow unit is 78 ± 9 mg/L. Denitrification occurs mainly in the alluvial layers where the occurrence of organic matter enhances the loss of nitrate by heterotrophic processes.

#### The Baix Fluvià aquifer

The unconfined aquifer of the Baix Fluvià consists on the upper sedimentary unit that constitutes the fluvio-deltaic system of the Fluvià River. Since most of the wells exploit such unconfined, 20-m-thick layer, no data were collected from the lower leaky aquifer. Recharge from the Fluvià River is also limited to the western reach. Mean nitrate concentration is 43 ± 7 mg/L.

### Climate change predictions for Catalonia: downscaling *T* and *P* values

Present climate projections derived from climatic/atmospheric models provide estimates for temperature (*T*) and precipitation (*P*) variations for the next decades. In most cases, downscaling processes are necessary to refine such predictions and to offer more appropriate data to hydrologists so future water budgets can be estimated with the predicted parameters. Calbó et al. (2016) calculated the seasonal and annual temperature and precipitation projection for 2021 and 2050 using distinct climatic databases (MERCAT, ESTCENA, EuroCORDEX, CMIP5, and DCPP), as well as distinct downscaling methods (dynamic and statistic), for the distinct geographical areas in Catalonia, namely, Pyrenees, inland areas, and coastal areas. Downscaling resolution varied according the method and the area of application, and it ranged between 0.04 to 0.2°. Such scaling approaches are necessary, especially for rainfall, as in areas such as Catalonia, the complex orography and land-sea contrast are largely misrepresented by global models.

The analyzed climate projections by Calbó et al. (2016) reveal a robust temperature rising trend over Catalonia for the coming decades over all geographical/climatic areas of Catalonia. Using the median from all downscaled datasets, the surface temperature will potentially increase by + 0.8 °C during the current decade (2021) and even reach + 1.4 °C by mid-century (2050), as compared to the reference period 1971–2000 (Table 2). The rising trends may be even more powerful for the Pyrenean region, particularly in summer. For rainfall, the climate projections show a decreasing trend, but with an uncertain slope. Indeed, variation of rainfall rate in the current decade is barely significant. By 2050, on the contrary, a decrease in rainfall is clear, with a median in distribution of estimated values around − 10% in spring, summer, and autumn. Average annual decrease in Catalonia is − 6.8%, being larger in the coastal areas (− 8.3%) and smaller in the Pyrenees (− 5.3%). These projections are based on moderate emission scenarios of radiative forcing (i.e., A1B, RCP4.5), and consequently, other estimated values associated with future climate change might be slightly higher than those herein introduced.

**Table 2:** Annual median temperature and rainfall variations for the 2021 and 2050 horizons in Catalonia with respect to 1971–2000. Data in parenthesis correspond to the 5th and 95th percentile. Data after Calbó et al. (2016)

	Climatic area:	Pyrenees	Inland areas	Coastal areas	Catalonia (surface weighted average)
2021	Δ*T* (°C)	0.8 (0.5/1.1)	0.7 (0.5/1.0)	0.7 (0.5/1.0)	0.8 (0.5/1.0)
	Rainfall (%)	− 0.2 (− 7.8/8.0)	0.7 (− 14.1/8.0)	− 2.4 (− 20.7/6.0)	− 2.4 (− 13.4/5.8)
2050	Δ*T* (°C)	1.6 (0.9/2.2)	1.4 (0.9/2.1)	1.4 (0.9/2.0)	1.4 (0.9/2.0)
	Rainfall (%)	− 5.3 (− 16.1/− 1.2)	− 6.5 (− 23.7/1.4)	− 8.5 (− 27.1/2.3)	− 6.8 (− 22.0/− 0.7)

### Water budget estimations for future climate scenarios

With the intention to estimate available water resources in the distinct basins of Catalonia under climate conditions predicted for 2021 and 2050, Mas-Pla et al. (2016) conducted a hydrologic water balance considering predicted rainfall to estimate precipitation inputs (*P*) and predicted temperature and present land use cover at a sub-basin scale to estimate the actual evapotranspiration (AET) using Zhang et al. (2001) approach. These estimations are based on predicted data for at least two meteorological stations within or closer to the sub-basin boundaries. The difference between these two terms (*P* and AET) provide the amount of available water resources as surface water and groundwater; in other words, the amount of “blue water” in contrast to “green water” (or evapotranspirated water), according to the intuitive terminology stated by Falkenmark and Rockström (2004).

**Fig. 3:**
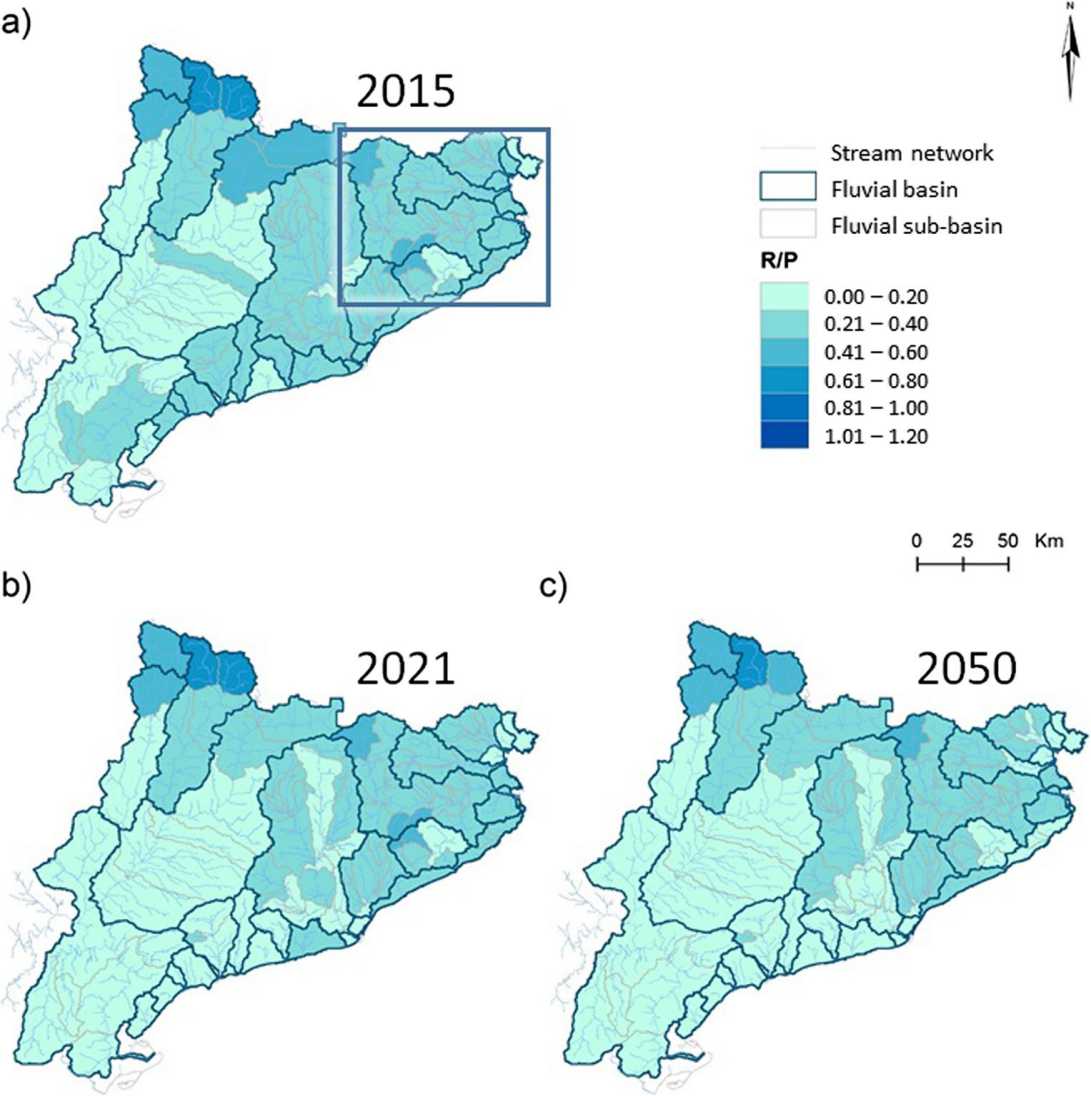
Regional distribution of the R/P ratio, as the ratio between available resources and precipitation, for each sub-basin including the inner basins and those of the tributaries of Ebro basin within (or partially within) Catalonia, at **a** present (2015), and the future scenarios at **b** 2021 and **c** 2050; after Mas-Pla et al. (2016). Rectangle in map (**a**) indicates the location of Fig. 1

Table 3 shows the mean ratio between available resources (or “blue water”) and the precipitation (*R*/*P*; where *R* stands for “available resources” and *P* for “precipitation”) in the hydrological basins of the aquifers considered in this study (Fig. 3). *R*/*P* values decrease in time from 2015 to 2050 pointing out the relative magnitude of the water loses in all the selected basins. Other recharge terms such as stream inflow to the aquifer and regional flow systems from the surrounding mountain ranges were not included for simplicity in the water budget approach. Nevertheless, the occurrence of these terms will later on be recovered for the discussion of nitrate fate under changing climatic conditions. It must be stated, however, that *R*/*P* estimations given in Table 3 and Fig. 3 assume uniform land uses for the first half of the twenty-first century. It is expected that agricultural areas will remain active in the plain areas and that some afforestation may happen in the hills or mountain areas as a result of crop abandonment. In this case, expected changes will be minor and, especially at higher altitude zones, they would represent an increase in evapotranspiration and, therefore, a diminution of available resources. Consequently, predicted values for 2050 shown in Table 3 and Fig. 3 could be slightly overestimated as a result of excluding such land use variations.

**Table 3:** Summary of available resources, as *R*/*P*, according to published data

		Osona	La Selva	Onyar	Garrotxa	Baix Ter	Baix Fluvià
*R*/*P*—2015^a^		0.307	0.202	0.292	0.380	0.238	0.216
*R*/*P*—2021^a^		0.262	0.188	0.278	0.357	0.229	0.208
*R*/*P*—2050^a^		0.256	0.165	0.254	0.338	0.206	0.186
Average GW Rec, *R*(gw)/*P*^b^		0.100	0.120	0.150	0.170	0.150	0.150
2015	*R*(sw)/*P*	0.207	0.082	0.142	0.210	0.088	0.066
2021	*R*(sw)/*P*	0.162	0.068	0.128	0.187	0.079	0.058
2050	*R*(sw)/*P*	0.156	0.045	0.104	0.168	0.056	0.036

*sw* surface water, *gw* groundwater

^a^Mas-Pla 2018

^b^Alcalá and Custodio (2014)

A main difficulty for a basin scale analysis is separating between surface water and groundwater resources for the predicted *R*/*P* values. No specific estimations are available for the percentage of infiltrating groundwater from the total resources. Moreover, varying land cover distribution may introduce a large uncertainty of any potential estimation. In consequence, and for the sake of discussing climate change effects on nitrate polluted aquifers, some average values will be used. In this sense, Alcalá and Custodio (2014) mapped the average recharge in continental Spain based on the atmospheric chloride mass balance, after a geostatistical analysis at a discretization grid of a 10 km × 10 km. Average groundwater recharge values for the selected aquifers, as estimated by Alcalá and Custodio (2014), are also included in Table 3.

Regarding nitrate, the proportion between runoff and infiltration is important to determine nitrate leaching to groundwater. Soil parameters controlling the water balance, such as soil texture, organic content, hydraulic conductivity, wetting front pressure, field capacity residual water content and porosity, as well as ion exchange capacity, are key (Vachaud and Chen 2002; Cameron et al. 2013), and they vary with time according to land use variations. These processes, yet relevant at a local scale, can hardly be included in a basin scale analysis.

## Climate change effects on groundwater quality

### Hydrological concerns

As introduced, this paper aims to discuss climate change effects on groundwater quality, taking nitrate as a paradigmatic pollutant, based on the effects of the regional water balance. For the next decades, major changes on land use and groundwater withdrawal rates are not expected. This will be the case for all the regional aquifers herein considered. Consequently, variations driven by natural factors—in particular, those induced by climate changes—and those forced by maintained or increasing human demand will modify the basin water budget. Ecological demand, as the amount of water needed to maintain natural habitats and ecosystem services, will also depend on water availability and on environmental transformation, and it will create a conflict with human interests in a context of water reduction.

As pointed out from climate predictions, *resource reduction will primarily affect direct groundwater recharge and surface runoff*. Surface runoff is relevant when discussing nitrate in alluvial aquifers since stream-aquifer interaction is the main source of low-nitrate content input to groundwater. This is particularly relevant at loosing stream reaches whether under natural gradient conditions or capture by discharging wells. In this sense, what presently constitute a recharge pole in the alluvial fluvio-deltaic aquifers of the Baix Ter and Baix Fluvià systems will surely reduce its contribution in the near future. If the consequences of diminishing stream discharge in the lower reaches of a river basin are to be examined, a set of outcomes can be enumerated. Namely, a stream discharge reduction implies several hydrological effects: (1) less instream flow, (2) less aquifer recharge in the loosing reaches, (3) a diminution of the water table of the unconfined aquifer, and finally (4) a reduction of the flow discharging back to the river in the gaining stream reaches or across other aquifer boundaries. Notice that those repercussions will worsen in all these areas by the predicted lower rainfall input and because of higher temperatures, more evapotranspiration in crops and forested land, especially during the summer season (Calbó et al. 2016).

Nevertheless, rainfall recharge through infiltration stands as the main input component of nitrate to the subsurface. Direct groundwater recharge effects on nitrate aquifer concentration can be examined using the previously introduced nitrate mass balance model. Based on such gross approach that assumes complete instantaneous mixing, the evolution of nitrate concentration is delineated for a 50-year period. The model assumes that runoff is nil in alluvial plains. Then, rainfall recharge in the Onyar, Garrotxa, Baix Ter, and Baix Fluvià alluvial aquifers equals the full R/P ratio derived from the water balance (Table 3). For the Osona and Selva basin cases, only a fraction of the available resources defined by R/P would infiltrate, and this fraction is given by the chloride balance, which is assumed constant for future hydrologic scenarios.

**Fig. 4:**
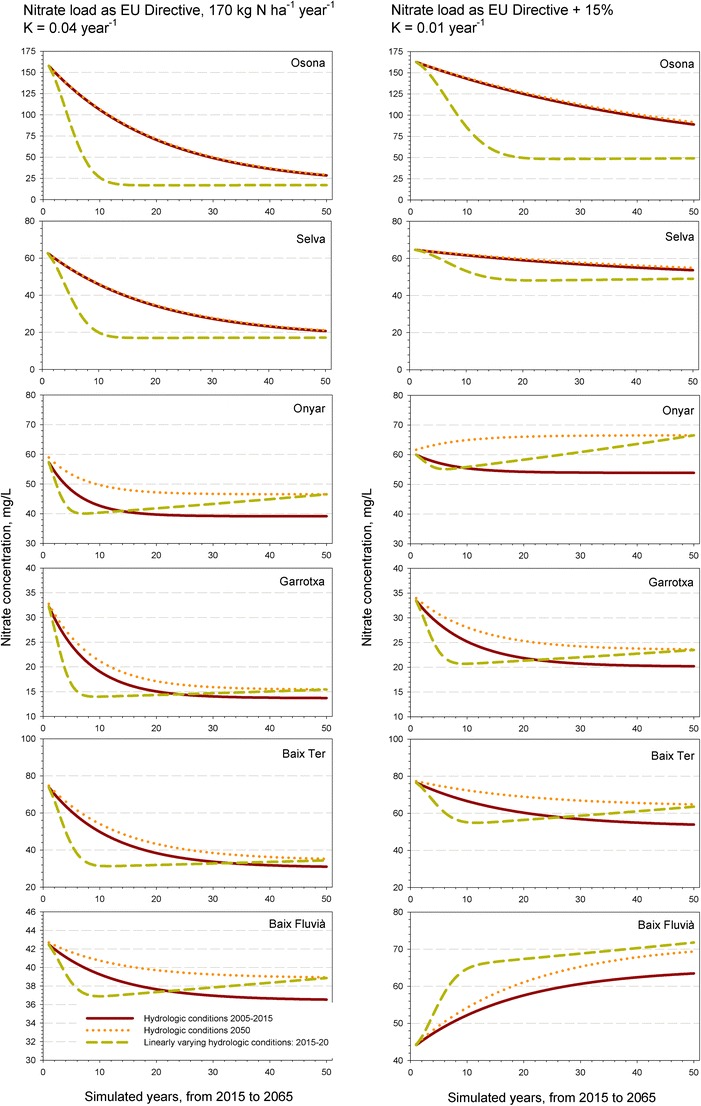
Evolution of nitrate concentration in the aquifer based on the mass balance model for the distinct aquifers considered in this study, under different future hydrological conditions (present, 2050, and a linearly variation of recharge rates), and considering two simulation scenarios according to nitrate input load and decay rates

Nitrate concentration evolutions based on the mass balance model (Fig. 4, left column) are determined by each aquifer turnover time (*t*_*d*_ = *V*/*Q*), the initial concentration (i.e., the mean value obtained from field data), and the total load estimated accordingly to the maximum fertilization rate, as allowed by the EU Directive, minus the nitrogen crop uptake (as estimated in Appendixes 2 and 3). All hydrological systems show a decline of the nitrate concentration towards the estimated final equilibrium value, $\overline{W}/Q\left(1+K{t}_d\right)$. Given their high initial concentration, recharge under future hydrological scenarios will decrease present groundwater nitrate concentration despite a reduction of the total rainfall recharge. Such counter-intuitive outcome indicates that hydrological dynamics of the system will naturally decrease pollution levels despite a loss of the dilution capacity, as long as input loads are kept at EU Directive levels.

A significant difference is observed between simulations (Fig. 4, left column). Those evolutions based on the hydrologic conditions (i.e., R/P) set for 2005–2015 and 2050 show a continuous decline towards the equilibrium value that is reached earlier in those aquifers with small turnover times. This is because both *R* and *P* values are assumed constant, and only the magnitude of *P* varies according to climatic predictions affecting *Q* and, consequently, the final equilibrium concentration. Complementarily, a linearly varying change of hydrologic conditions for the next 50 years implies a faster achievement of the nitrate concentration equilibrium value.

The cases of the Onyar and Fluvià River alluvial aquifer, and those of Garrotxa and Baix Ter aquifers to a minor extent, offer a more complex response as an initial quick decrease of nitrate concentration is counter-balanced by a later increase (Fig. 4, left column). The final concentration, however, always tends to the equilibrium value set by the 2050 conditions. This behavior is controlled by varying annual recharge rates that annually modifies the initial concentration under a constant nitrogen load. The annual modification of the initial concentration produces a shifting of the nitrate evolution trend after the first years of simulation. Despite such a potential response, a tendency to the equilibrium nitrate value set by the hydrological conditions in 2050 must be expected.

This outcome points out the relevance of input nitrogen loads as a managing term to control nitrate concentration especially under scarcity conditions. In the cases of Osona and Selva basins, conversely, the assumption of instantaneous mixing can hardly be sustained given the regional extent of both hydrogeological systems. Therefore, such a concentration reduction must be considered as a potential, indicative event.

As expressed by the analytical solution (Eq. A1-6), such a common decrease of nitrate content is due to the large initial concentrations as compared to the term $\overline{W}/Q\left(1+K{t}_d\right)$. The question is why such high average nitrate concentrations have been reached upon the past decades. Aside of distinct hydrological conditions, we assume that the load $\overline{W}$must have been larger than the legal limit of 170 kg N/ha/year used in the simulations for many decades, plus the use of chemical fertilizers that have not been included in the computation of the annual applied nitrogen load in Appendix Table 4.

A second simulation considers a + 15% increase of nitrate load and a reduction of the decay factor to 0.01 year^−1^ to force even more unfavorable conditions (Fig. 4, right column). Under these circumstances, the aquifer of the Baix Fluvià and Onyar River reverse their evolution trend to show an increase of nitrate concentration, indicating that total nitrate load is indeed a key factor. The type and magnitude of the nitrate trend is determined by fertilization practices and the use of manure management technologies and policies (Burton and Turner 2003), and it should be kept as a key factor when dealing with adaptation strategies. As a rule of thumb, the mass balance model points out that *a reduction of the actual nitrate load proportional to recharge variations in a scenario of no decay should be sufficient to avoid a worsening of groundwater quality*.

As shown, hydrological aspects as well as fertilization rates control nitrate evolution. A most efficient use of fertilizers must be sought to reduce nitrogen inputs, as well as other ions to groundwater (Menció et al. 2016). Nevertheless, crop production expectations, and more importantly the utilization of manures and slurries as fertilizers, as a means to reuse a waste product of livestock rising, will require an adjustment between nitrogen applications and environmental nitrogen assimilation/removal rates under future scenarios. Such balance is difficult to estimate, and even more difficult to implement as an environmental policy, yet they have a relevant influence on the final nitrate concentration as shown by the lumped-parameter model. Good agricultural practices devoted to limit nutrient applications will need to be restrictively applied to insure a decrease of nitrate content in groundwater. Efforts to reduce such inputs, even below the legal amounts set by the Nitrates Directive, by promoting alternative treatment of organic fertilizers are paramount to alleviate the expected impact derived from changing hydrological conditions.

In summary, this mass balance approach can be realistically taken as a broad estimation of what may happen in the future. From a management perspective, it provides an interesting unbiased contribution as it states that managing fertilization stands as the most effective action to insure that climate change will not worsen nitrate concentrations. Despite future scenarios show a general decrease in nitrate for 2050, the sensitivity of the proposed approach suggest that further modeling is needed to more accurately assess the impact of climate change, as well as tailor more specific solutions to nitrate pollution, especially for local smaller aquifer units. As shown in Fig. 4, turnover time and initial concentration stand as the factors that determine the aquifer vulnerability under a constant input load; therefore, the outcome of the model will be more credible when both parameters are strictly estimated for a given hydrogeological unit.

Two additional hydrological facts must be conceptually considered in addition to the above discussion. On one hand, decreasing stream runoff will alter the magnitude of surface water recharge. This is relevant in the cases of all analyzed alluvial aquifers. On the other, the overall hydraulic gradient will also be flattened due to a decrease of the inland hydraulic head at the boundaries, at the stream itself or at the coastline. Sea level rise has been measured at a rate of + 3.9 cm/decade, in the Baix Ter shore (Martín-Vide et al. 2016). Resulting lower hydraulic gradients will also reduce outflows towards streams and wetland, consequently reducing the rate of nitrogen assimilation that takes place in riparian areas.

A decrease in total recharge implies an increase of the turnover time of groundwater within these hydrogeological systems. Denitrification processes will depend on the subsurface chemical environment, so increasing turnover time might benefit nitrate transformations where subsurface environmental conditions are appropriate. Usual heterotrophic denitrification linked to the occurrence of organic matter and lack of oxygen can certainly be modified. Oxygen solubility will drop at higher temperatures, yet subsurface thermal conditions will eventually remain nearly unchanged at a given depth. Bonnett et al. (2013) report that the temperature response of denitrification rate was exponential, yet it also increased under nitrate-rich infiltration flooding conditions. Nevertheless, nitrogen isotopes for the two alluvial aquifers (Baix Ter and Baix Fluvià) indicate that, under present conditions, only one out of four samples show some degree of denitrification (Table 1), which suggests that even though conditions turned to be favorable, nitrate degradation would not be a dominant nitrogen loss process.

Water scarceness will hence largely affect alluvial aquifers and their nitrate content. Large hydrogeological systems, as the Selva basin, may even reduce their concentrations as shown by the simulations (Fig. 4). However, the fact that they are also recharged by large-scale regional groundwater flows originated from the surrounding ranges (usually without agricultural land uses and therefore with a very low-nitrate content) or even from the basin basement provides and additional diluting flow. The reducing conditions of these deep flows locally enhance natural nitrate attenuation (Puig et al. 2013). In consequence, they will be better off than those that just depend on local inputs (i.e., stream recharge and rainfall recharge). These large-scale regional flow systems store and transmit groundwater to the adjacent aquifers systems. From a groundwater quality perspective, their contribution alleviate nitrate content in the deep aquifer levels which, as in the case of La Selva basin, presently show a lower nitrate content than the shallow unconfined aquifers (Table 1 and Fig. 4). Regional flows additionally have a second advantageous factor: they will unceasingly contribute to the recharge of other systems, usually located in basins, as mountain areas are expected be more resilient to climate change effects on groundwater recharge, as infiltration rates in mountain areas is permeability-limited rather than recharge-limited due to the thin soil layer overlying low permeability rocks (Flint et al. 2008; Meixner 2016). These regional flows balance the loss of surface rainfall infiltration in low altitude areas. Such flows, as in the Selva and Baix Ter alluvial plains, presently contribute with a high-quality recharge, and it should be expected to remain so for the next decades (Vilanova et al. 2008; Boy-Roura et al. 2018).

Exploitation regimes will become a relevant term of the water balance as well. Although among the selected aquifers only the Baix Ter system can be qualified as over-exploited, all of them usually recover their hydraulic head after the irrigation season due to rainfall, stream, and regional flow system contributions, being the later particularly relevant in the Selva and Osona basins. Alluvial aquifers need to be distinguished again because of the influence of well capture zones on the stream discharge. Induced stream infiltration may end up by drying creeks and river reaches, as it presently occurs in the Onyar River and Baix Fluvià River aquifers, representing a cease of low-nitrate content waters into the aquifer and causing severe ecological impacts. Depleted groundwater recharge in future climatic scenarios and similar withdrawal rates will suppose a stronger pressure on each alluvial aquifer system, as turnover times will decrease due to a diminution of the stored water resources. Confined aquifer groundwater resources in fluvio-deltaic formations would be less prone to be affected by climate change, provided that their recharge is associated to regional flow systems, usually with a participation of basement-originated groundwater upward flows as in the Baix Fluvià and Baix Ter aquifers. However, leaky aquifer layers linked to unconfined aquifers that support agricultural activity will present a higher risk of quality resources deterioration.

From a water resources management perspective, two actions can be adopted to minimize the effects of climate change associated to the hydrological unbalance that will represent maintaining a similar demand under drier conditions. First, *allocate exploitation wells in places and aquifer units that will be more resilient to recharge variations*, to exert a minor pressure for adjacent aquifers and streams. Second, *reduce groundwater exploitation rates* by increasing agricultural efficiency, urban water saving actions, and diminishing losses in the distribution network and, ultimately, *reclaim treated urban wastewater as an effective alternative to compensate water scarcity* through, for instance, managed aquifer recharge (e.g., Dillon et al. 2010).

The future role of confined aquifers, as presumably more resilient water bodies under changing hydrological conditions, has already been mentioned in this analysis. Besides, nitrate concentrations are usually low in these formations, and they can provide good quality freshwater resources, as those in the Baix Fluvià system. However, leaky aquifers, as the Baix Ter area, may present nitrogen under the form of ammonium due to the reduced conditions in depth (Mas-Pla et al. 1998). Nevertheless, the long-term response of these hydrogeological systems to climate change is uncertain in terms of sustainability, and their quality can be influenced by naturally geological occurring elements, such as arsenic and fluoride as in the Selva basin. Groundwater outflows from range areas to the confined layer of the nearby basins, usually enhanced by fault zones, stand as valuable resources that have to be quantified for each case to avoid over-exploitation (Folch and Mas-Pla 2008). Indeed, such flows are of certain relevance, at smaller or greater extent, in most of the selected aquifers; yet only in La Selva basin they entirely balance the intensive groundwater withdrawal that takes place during the irrigation season.

Deep confined or leaky aquifers in fluvio-deltaic plains, such as the Baix Ter and Baix Fluvià areas, should be treated differently as their recharge mostly originates in the inland part of these recent sedimentary units. Formed during the late Pleistocene and Holocene eustatic changes, these lower hydrogeological units are in hydraulic contact with the uppermost unconfined aquifer layers along the inland boundary. As expected, such confined/leaky aquifers would not be as resilient to climate change as those in continental locations, as La Selva, Osona, and Garrotxa systems. They will be susceptible to recharge rate reduction and to nitrate enrichment by downward vertical percolation due to held (or increased) exploitation rates.

Wastewater reclamation and management rises as a most convenient and reliable local alternative resource. It may not be of direct use in mountain areas, or in the basin headwaters, where the reduction of available resources will affect more the availability to provide downstream discharge than to supply local demand. Outflows from treatment plants dumped into rivers will partially compensate decreasing stream discharge, and despite introducing nutrients into surface water, no major quality changes should be envisaged. Contrarily, water reuse in lowland and coastal areas stands as a mandatory premise for hydrological planning. Alluvial plains with intense agricultural water demand are potentially the first allocation for treated wastewater, at least for specific uses as the total treated water volumes will be by far smaller than the total agricultural demand. Environmental uses, as wetland restoration and maintenance, of reclaimed water are interesting alternatives as well, and they have been successfully used in the preservation of the natural area of the Empordà wetlands, north from the Baix Fluvià area. Both agricultural and environmental uses might benefit from the nutrient content of reclaimed water; yet in excess, they might affect habitat equilibrium and enhance eutrophication. Moreover, treated wastewater applied as irrigation will sum up its nitrogen content with that from manure resulting in an excess load that will concern the final soil and groundwater nitrate concentration.

### Environmental concerns

In the effort to protect streams and wetlands as valued groundwater dependent ecosystems, water balance variations must be taken into consideration. On one hand, stream dilution will not be as effective; on the other hand, human and groundwater inputs would also increase the stream and wetland nitrate content (Ducharne et al. 2007). Nitrogen, as a nutrient, is highly assimilated by plants in riparian areas and pond boundaries, and such process may prevent eutrophication in environmental healthy habitats (McMahon and Böhlke 1996; Smith et al. 1999; Galloway et al. 2003; Smolders et al. 2010).

Assuming a recommended rate of fertilization (i.e., manure application twice a year at the established rates of 170 kg N/ha/year in vulnerable agricultural zones and 210 kg N/ha/year in other areas; EU Nitrates Directive), nitrogen mainly leaches from arable soils between summer and winter crops, just after manure application when plant demand is low. Soil accumulated nitrogen mass after decades of continuous high fertilization rates will provide unceasing inputs towards the water table that may increase after intense rainfall events in unconfined aquifers. In this sense, Boy-Roura et al. (2013b) point out that nitrate content of the natural springs in Osona was uniform along the whole year, while other physico-chemical parameters varied following a fortnight periodicity monitoring of 13 springs during a year. This fact was interpreted as the effect of accumulated nitrogen, as nitrate in soils and the upper aquifer levels; consequently, the unvarying nitrate content observed in springs can be compared to the concentration reaching the unconfined aquifer and percolating to its deepest levels. Based on groundwater flow and transports simulations in Canada, Paradis et al. (2016, 2017) also pointed out that the progressive attainment of steady-state conditions related to present day nitrogen loadings will increase nitrate in groundwater for the next decades and that a changing of load rates will not immediately be noticed in the sampling wells due to the distinct behavior of each hydrogeological formation. In this sense, if rainfall recharge and river inflow are predicted to decrease larger nitrate concentrations might occur as the total nitrate flow mass will depend on the new infiltration rate across the unsaturated zone and the new hydraulic gradient, in its magnitude as well as in direction, within the aquifer. In other words, *changing hydrogeological dynamics as a response to water balance variations will influence the overall nitrate content and fate in the subsurface, as well as their impact on groundwater dependent ecosystems*. This effect on the nitrate aquifer content, however, must be understood in the context of the mass balance model outcome (Fig. 4).

Back to the model results depicted in Fig. 4, soil-stored nitrogen contributes to the net annual nitrogen mass that percolates to the unconfined aquifers. In such case, the final load value, *W*, will be larger and nitrate content within the aquifer may tend to rise as in the Baix Fluvià aquifer.

Pumping rates will additionally interfere in this dynamic scheme. As mentioned, capture zones affecting stream reaches and wetlands may progressively dry them up. Recovered natural flow fields after the irrigation season feed groundwater-dependent stream reaches and wetlands again, as in the Baix Ter and Baix Fluvià areas (Mas-Pla et al. 1999). This is the reason why, especially in wetlands and ponds, preserving the natural habitat in their shores, where nitrogen assimilation/immobilization processes occur, is so essential.

Soil processes, such as nitrogen mineralization, immobilization, and percolation, depend on the soil characteristics including organic matter abundance, pH, microbiological activity, and, specifically, temperature and humidity, being organic matter inversely proportional to temperature (Leirós et al. 1999). As reviewed by Stuart et al. (2011), chemical processes under new conditions of soil temperature and, principally, humidity will affect mineralization and leaching: soil moisture appears to be the primary variable affecting soil enzyme activity (Sardans et al. 2008), and nitrogen mineralization and nitrification are related to temperature and indirectly to rainfall (Rustad et al. 2001; Emmett et al. 2004). In its turn, microbial activity is lowest when the soil is either dry or saturated, and increasing summer droughts will reduce mineralization and N and C fluxes whereas increasing summer precipitation could enhance losses (Borken and Matzner 2009; Dirnböck et al. 2016). In summary, complex processes will determine nitrogen mass flow to the aquifer. However, it is clear that such mass flow will not cease in the future decades if continuous fertilization occurs. *Never can the occurrence of attenuation processes as denitrification, yet active in some aquifers, be considered as an acceptable solution* because it would put aside true management decisions, and it depends on a natural process that might vary spatially and/or temporally under varying hydrogeological dynamics.

### Economic, social, and political concerns

Beyond hydrological and environmental concerns, climate change issues associated with nitrate pollution have strong economic and social sides. It is obvious that groundwater high nitrate concentrations are related cattle rising (mainly swine), being a crucial development activity in rural areas and for the region’s economy. More specifically, in the case of Catalonia, pork meat production reached a total value of 6.17 kM€ in 2013, being a 33% of this amount derived to foreign exportation (Ramis and Pérez 2016). Swine manure has been used as fertilizer for several decades and, as seen, high nitrate concentrations are presently found without showing any clear decreasing tendency despite the use of agricultural good practices. Given the importance of this economic sector, it is not plausible that manure production and use will decrease in a medium run and, in consequence, arable lands will be fertilized at its maximum capacity to get rid of manures and slurries. As in any pollution case, input control is a key factor in controlling the spread and evolution of the pollutant, as already discussed for the model results (Fig. 4). Alternative nitrate treatment processes should thus be encouraged to be used (Seidel et al. 2011), yet at high and undesirable costs that may only be affordable to warrant domestic supply. In the context of climate change, as mentioned, lower recharge in the main agricultural areas located on alluvial aquifers will hamper dilution. In this sense, the problem becomes twofold: water scarcity and pollution.

So far, the nitrate pollution control under climate change faces two main challenges: (1) access to appropriate groundwater quality for human uses and (2) preventing the environmental deterioration of groundwater bodies and linked ecosystems. To cope with them, necessary actions to overcome water resources scarcity and its present poor quality will need additional investment as well as a careful planning.

Such additional investment can be clearly considered as a climate change adaptation cost, involving allocating groundwater and alternative water resources, such as reclaimed water, and new infrastructure construction. Should actions (and their costs) depend on the sole decisions of well owners, a disorganized modification of the actual exploitation habits is likely to occur, leading to a new scenario far from desired sustainability goals. Therefore, *institutional and government involvement should be active, committed, and comprehensive, so all agents are involved in the adaptation process* (Pla et al. 2017). In this sense, potential actions to execute are (1) substituting well locations for others that will allow a sustainable supply by avoiding over-exploitation (whether by exhausting resources, deteriorating quality, affecting ecosystems, or being economically unfeasible; Custodio 2002), (2) increasing the efficiency of the existing water distribution networks, and (3) building up new ones for reclaimed water use. Facing nitrogen input control, implementing good agricultural practices, especially on manure application, implies setting a maximum number of animal heads whose manure can be conveniently assimilated as fertilizer. This may, of course, limit economical profits, but enhance environmental benefits. Applying alternative manure treatment facilities as a means to reduce nitrogen arrival to the environment should then be taken. Irrigator communities (or water user’s associations, where actors are diverse), such as that existing in the Baix Ter area, stand as legal and binding agents necessary to achieve proper adaptation strategies (Garrido and Llamas 2009; Re 2015; Re et al. 2017).

Investments need social and political support. From the *social perspective*, population needs to understand the needs of such actions and the fact that under the new climatic scenarios the cost of good quality water resources will increase. From the *socio-political side*, education focused to understand the value of a scarce resource should be oriented not only to citizens but also to farmers and, especially, to first-sector investors whose profits are presently based on water resource managing strategies that would be unappropriated for the next decades. The livestock sector has to face it. From the *political responsibility*, a commitment is needed to impulse directives and measures focused on water resources planning that recognize the urgency for actions. The water balance approach used to discuss the effects of climate change on the quality of groundwater resources indicates that the “nitrate problem” may become even worse if the no-action attitude prevails. Active political involvement is paramount to force action and to adjust government as well as private responsibilities on preserving groundwater quality resources, explicitly recognizing the environmental duty of private investors and farm owners that are relevant actors on the proper setting of management solutions.

In this sense, scientific-based opinions must be explained to stakeholders and decision makers so groundwater quality issues are included in their agendas; so the necessary economical investments and actions devoted to face nitrate concentration increases because of varying water balances can be properly accomplished. Such actions, as well as new water supply alternatives, must be sought and scheduled, so they can be appropriately programmed and executed in its due time and their *future costs included in today’s operational budgets so to avoid considering them as externalities when they will be urgently needed*.

## Concluding remarks

The examination of the climate change effects on the water balance in distinct aquifers, such as those taken as reference in this contribution, points out that water scarcity, pollution inputs, and management strategies are the key issues on coping with adaptation actions oriented to control nitrate groundwater pollution in the next decades. Water scarcity, controlled by climatic factors, can be counter-balanced by an efficient water use and the use of non-conventional resources, such as reclaimed wastewater. The fact that recharge rates will decrease in western Mediterranean areas will threaten the success of dilution processes. Nevertheless, given present concentrations in groundwater there is no certainty that the overall groundwater nitrate content will likely be enriched as an appropriate magnitude of the nitrogen inputs could counterbalance the effect of diminishing recharge. Therefore, appropriate manure management will thus be sufficient to control nitrate pollution. Conversely, those hydrogeological units additionally recharged by regional, large-scale systems will be more resilient and keep the present dilution rates during the next decades. From a hydrochemical perspective, climate effects on nitrogen mineralization will be variable, although drier conditions might reduce nitrate formation.

Even though simulated trends points out a future decline of nitrate content in groundwater, this hydrological analysis makes clear that controlling nitrogen inputs originated from fertilization stands as the key point in management strategies, especially in alluvial and fluvio-deltaic aquifers with small turnover times. This statement is already considered by good agricultural practice programs and the analysis of climate change effects that arise from the mass balance model indicates that just a no-action response, keeping business as usual, is not necessarily an erroneous option. Given the broadness of the approach, however, it is wise advising that watershed hydrological plans, together with development policies, must stress fertilizer reductions, as well as act on the livestock farming industry as an inescapable issue to avoid a further deterioration of the present groundwater quality status.

Environmental preservation, especially of groundwater dependent ecosystems (riparian areas and wetlands), is essential to maintain nitrogen assimilation rates. New climate scenarios, as well as higher nitrate content in groundwater, may contribute to transform (and possibly degrade) habitats. Groundwater quality control and environmental monitoring must be maintained with seasonal periodicity to track expected changes. Regional planning, including agricultural, environmental, and water related issues, must be flexible enough to adapt to changes as soon as they become evident. Unplanned actions, left to the landowner’s initiative to satisfy private water needs, will jeopardize the achievement of a sustainable water management plan with irreversible damages. Socio-economic considerations do finally round out the complex decision-making process to deal with climate change and groundwater pollution. Whatever the water balance changes predicted for the next decades, noticeable effects might be noticed soon, and their impacts on groundwater quality are expected to persist in the environment for actually long periods.

## Appendix 1. Approximation to the aquifer response to inputs: a nitrate mass balance, lumped-parameter model

Mass balance approaches provide a means to evaluate the climatic variability effects on nitrate concentration in aquifers. These models assume that diffuse inputs on the aquifer surface are completely mixed horizontally and vertically, which is reasonable based on the large extent of the agricultural land use compared to the aquifer thickness. Such a gross approximation permits a quick and useful estimate of the behavior of these systems without creating elaborated numerical flow and transports models. They also deliver some clues for groundwater managing under a changing hydrological environment.

The approach used herein is based on the solutions proposed by Thomann and Mueller (1987, Chapter 4.2) for a lake as a completely mixed system. A mass balance equation for an aquifer, as a likewise system, is given by

A1–1 $$ \frac{d(Vc)}{dt}=W(t)- Qc- KVc $$

where *V* is the aquifer water volume [L^3^]; *c*, the concentration of the diffuse source [M L^−3^]; *W*(*t*), the total mass per unit time entering the system by diffuse pollution [M T^−1^]; *Q*, the natural outflow of the aquifer [L^3^ T^−1^]; and *K*, the first-order decay coefficient for reactive compounds [T^−1^]. The component *Q* can also be assimilated to the aquifer recharge, mainly from rainfall, that also exits the system during a given time period without any change of the stored water mass; i.e., *dV*/*dt* = 0. Other inputs, such as inflow from rivers and irrigation returns, could also be considered, yet their magnitude will need a full flow model which is beyond the interest of this estimation.

Expanding the derivative of the left-hand side, and considering *dV*/*dt* = 0, Eq. (1) can be rewritten as

A1-2 $$ V\frac{dc}{dt}+\left(Q+ KV\right)c=W(t) $$

Taking *W*(*t*) as the system mass input, its solution is *c*(*t*) given *V*, *Q*, and *K* as constants. The complementary solution of Eq. (2) is given by *W*(*t*) = 0, meaning that the pollution input is nil and that the initial concentration within the aquifer (*c* = *c*_0_, at *t* = 0) will gradually decrease according to the flushing (dilution) capacity of the aquifer and the solute decay rate. A most desirable scenario, is not it? Under these conditions, the solution of Eq. (2) is given by

A1-3 $$ c={c}_0\exp \left[-\left(\frac{Q}{V}+K\right)t\right]={c}_0\exp \left[-\left(\frac{1}{t_d}+K\right)t\right] $$

where *t*_*d*_ = *V*/*Q* is the aquifer turnover or mean residence time (Maloszweski and Zuber, 1982; Kazemi et al. 2006, p. 23).

A particular solution, that fits with pollution derived from fertilization practices, is given by the “step input” which forces the input to a constant magnitude after a time *t*_0_

$$ W(t)=0,t<{t}_0 $$

$$ W(t)=\overline{W},t>{t}_0 $$

The solution of the step response is

A1-4 $$ c=\frac{\overline{W}}{Q+ KV}\left\{1-\exp \left[-\left(\frac{Q}{V}+K\right)t\right]\right\}=\frac{\overline{W}}{Q\left(1+K{t}_d\right)}\left\{1-\exp \left[-\left(\frac{1}{t_d}+K\right)t\right]\ \right\} $$

which tends to a steady concentration value, $c=\overline{W}/\left(Q+ KV\right)$, after a sufficient length of time has elapsed.

The total response, given the occurrence of an initial concentration *c*_0_ in the aquifer, is given by the sum of Eqs. (3) and (4)

A1-5 $$ c=\frac{\overline{W}}{Q\left(1+K{t}_d\right)}\left\{1-\exp \left[-\left(\frac{1}{t_d}+K\right)t\right]\ \right\}+{c}_0\exp \left[-\left(\frac{1}{t_d}+K\right)t\right] $$

Eq. (5) can be rearranged as

A1-6 $$ c=\frac{\overline{W}}{Q\left(1+K{t}_d\right)}+\left({c}_0-\frac{\overline{W}}{Q\left(1+K{t}_d\right)}\ \right)\exp \left[-\left(\frac{1}{t_d}+K\right)t\right] $$

illustrating that for scenarios when ${c}_0>\overline{W}/Q\left(1+K{t}_d\right)$ concentration in the aquifer will progressively decrease as the step input is not large enough to increase the overall aquifer concentration and at large times will achieve the equilibrium value, $\overline{W}/Q\left(1+K{t}_d\right)$. Otherwise, when ${c}_0<\overline{W}/Q\left(1+K{t}_d\right)$, concentration in the aquifer will continuously buildup until the equilibrium concentration is reached.

In the analysis of climate change effects, recharge reductions will affect the *Q* term, while fertilizer management and control will relate to the $\overline{W}$ term.

## Appendix 2

**Table 4:** Aquifer, land use, and hydrological characteristics of the studied hydrogeological systems used in the nitrate mass balance model

		Osona	Selva	Onyar	Garrotxa	Baix Ter	Baix Fluvià
Area	km^2^	316	670	41	57	96	64
Crop area	km^2^	225	450	37	52	86	59
Rainfall, *P*	mm	648	575	703	927	600	559
Rainfall, *P*	hm^3^	204.8	385.3	28.8	52.8	57.6	35.8
Depth	m	60	80	12	30	22	18
Porosity	[−]	0.10	0.08	0.12	0.12	0.13	0.15
Groundwater resources	hm^3^	1896	4288	59	205	275	173
Recharge ratio, *R*/*P* (2015)	[−]	0.100	0.120	0.292	0.380	0.238	0.216
Inflow/outflow, *Q* (2015)	hm^3^	20.48	46.23	8.42	20.08	13.71	7.73
Average initial concentration, *c*_*o*_	mg/L	165	65	61	35	78	43
Turnover time, *t*_*d*_ = *V*/*Q*	year^−1^	92.6	92.8	7.0	10.2	20.0	22.4
Excess nitrate	%	0.096	0.1085	0.1515	0.099	0.1163	0.1201
Nitrate load, *W*	kg NO_3_/year	1,626,171	422,014	3,675,825	387,571	752,993	533,467

Comments:

• Average aquifer mean parameters (depth, porosity) are taken from hydrogeological reports and field data.

• *R*/*P*—ratio among available water resources (*R*) and rainfall (*P*) taken as total recharge ratio in the mass balance model for alluvial aquifers

• The aquifer inflow/outflow rate is estimated as the product of rainfall magnitude times the *R*/*P* value.

• Average crop nitrogen uptake is estimated using the values given in Appendix 3.

• Total nitrate mass input considers the maximum allowed nitrogen mass per year by the EU Nitrates Directive (170 kg N/ha/year), the crop area, and the average nitrogen uptake.

## Appendix 3. Estimation of the average crop nitrogen uptake

**Table 5:** Annual average values per crop type in the studied region

*n*	Annual average N uptake^a^	Annual average crop production^b^	Annual average N uptake	Annual applied N as manure	Annual N excess
Crop	kg N/tonne crop	Tonne/ha	kg N/ha	kg N/ha	kg N/ha
Cereal	29.0	4.95	143.6	170	26.4
Citric	3.5	22.20	77.7		
Forage	165.0	2.85	470.8	170	Deficit
Olive trees	15.0	1.35	20.3		
Potato	3.5	22.54	78.9		
Sunflower/rapeseed	47.0	2.15	101.2	170	68.8
Sweet and dry fruit	8.2	6.80	55.61		
Vineyards	7.0	7.23	50.6		

^a^Boixadera J, Sió J, Àlamos M, Torres E (2000). Manual del codi de bones pràctiques agràries. DARP, Generalitat de Catalunya, 59 pp

^b^Superfícies i produccions dels conreus agrícoles. Any 2016 (DARP): http://agricultura.gencat.cat

**Table 6:** Crop surfaces in hectare

County	Osona	Selva	Gironès	Garrotxa	Baix Empordà	Alt Empordà
Associated aquifer	Osona	Selva Basin	Onyar	Garrotxa	Baix Ter	Baix Fluvià
Cereal	14,616	6020	7814	3860	12,152	21,286
Citric	6	3	4	1	0	1
Forage	10,276	2303	2660	2656	4051	6795
Sweet and dry fruit	5	275	369	10	1274	1040
Olive tree	36	16	61	3	274	2185
Potato	56	118	6	19	19	14
Sunflower/rapeseed	708	437	2230	324	1047	2914
Vineyards	12	21	72	10	171	1923
Other crops	919	1057	749	496	889	1181
Total	26,634	10,250	13,965	7379	19,877	37,339

Crop surfaces are expressed per counties in the official annuaries. Similar percentages are attributed to the studied aquifer surfaces as they concentrate most of the agricultural activity

**Table 7:** Crop surfaces in %

County	Osona	Selva	Gironès	Garrotxa	Baix Empordà	Alt Empordà
Associated aquifer	Osona	Selva Basin	Onyar	Garrotxa	Baix Ter	Baix Fluvià
Cereal	54.88	58.73	55.95	52.31	61.14	57.01
Citric	0.02	0.03	0.03	0.01	0.00	0.00
Forage	38.58	22.47	19.05	35.99	20.38	18.20
Sweet and dry fruit	0.02	2.68	2.64	0.14	6.41	2.79
Olive tree	0.14	0.16	0.44	0.04	1.38	5.85
Potato	0.21	1.15	0.04	0.26	0.10	0.04
Sunflower/rapeseed	2.66	4.26	15.97	4.39	5.27	7.80
Vineyards	0.05	0.20	0.52	0.14	0.86	5.15
Other crops	3.45	10.31	5.36	6.72	4.47	3.16
Total	100.00	100.00	100.00	100.00	100.00	100.00
% AreacCereal + forage + sunflower/rapeseed	93.46	81.20	75.00	88.30	81.52	75.21

**Table 8:** Estimation of the percentage of nitrogen excess

Excess, as kg N/ha/year	16.32	18.44	25.76	16.83	19.76	20.42
Excess, as kg NO_3_/ha/year	72.26	81.66	114.07	74.54	87.53	90.43
Percentage of N excess, %	9.60	10.85	15.15	9.90	11.63	12.01
